# What pathology is an indication for vacuum-assisted biopsy?

**DOI:** 10.1186/bcr2998

**Published:** 2011-11-04

**Authors:** WMV Shuen, RJ Currie

**Affiliations:** 1 Peninsula Radiology Academy, Plymouth, UK; 2 Royal Devon & Exeter NHS Foundation Trust, Exeter, UK

## Introduction

Vacuum-assisted biopsies (VABs) are used for both diagnostic and treatment purposes. Currently there are no set guidelines in our department as to who should proceed to a VAB. The purpose of this study is to analyse the indication for a VAB, the upgrade or downgrade rate when compared with the initial core biopsy and the overall final outcome of these patients.

## Methods

A retrospective search of all VABs performed from 1 April 2009 to 31 March 2010 was identified. The indication for VAB, the initial core biopsy results and the vacuum biopsy results and final outcomes were recorded.

## Results

A total of 37 VABs were performed within the year. Two were for treatment excision of fibroadenoma. A total of 35 were diagnostic VABs. Three went straight to vacuum biopsies due to either a small lesion or suspicion for a recurrent malignancy. Of the remaining 32 cases, 21 were for indeterminate M3/U3/B3 lesions, five for clinical and pathological mismatch, three for further clarification of core biopsy result, two for staging the extent of the tumour, and one for inadequate core biopsy. Thirteen out of 32 (40.6%) were upgraded from the initial biopsy, 10 proceeded to further procedures. Six out of 32 (18.8%) remained the same grade, two (33.3%) required further procedure. Thirteen out of 32 (40.6%) were downgraded, five (38.5%) proceeded to further procedures.

## Conclusion

Our audit has shown VAB is useful in providing a definitive diagnosis in a range of breast pathology, in particular B3 lesions. We therefore strongly advise its use in cases of pathological uncertainly to save patients from further unnecessary interventions.

